# Exploring peptide/MHC detachment processes using hierarchical natural move Monte Carlo

**DOI:** 10.1093/bioinformatics/btv502

**Published:** 2015-09-22

**Authors:** Bernhard Knapp, Samuel Demharter, Charlotte M. Deane, Peter Minary

**Affiliations:** 1 ^1^Department of Statistics, University of Oxford, 1 South Parks Road, Oxford, OX1 3TG, UK and; 2 ^2^Department of Computer Science, University of Oxford, Wolfson Building, Parks Road, Oxford, OX1 3QD, UK

## Abstract

**Motivation:** The binding between a peptide and a major histocompatibility complex (MHC) is one of the most important processes for the induction of an adaptive immune response. Many algorithms have been developed to predict peptide/MHC (pMHC) binding. However, no approach has yet been able to give structural insight into how peptides detach from the MHC.

**Results:** In this study, we used a combination of coarse graining, hierarchical natural move Monte Carlo and stochastic conformational optimization to explore the detachment processes of 32 different peptides from HLA-A*02:01. We performed 100 independent repeats of each stochastic simulation and found that the presence of experimentally known anchor amino acids affects the detachment trajectories of our peptides. Comparison with experimental binding affinity data indicates the reliability of our approach (area under the receiver operating characteristic curve 0.85). We also compared to a 1000 ns molecular dynamics simulation of a non-binding peptide (AAAKTPVIV) and HLA-A*02:01. Even in this simulation, the longest published for pMHC, the peptide does not fully detach. Our approach is orders of magnitude faster and as such allows us to explore pMHC detachment processes in a way not possible with all-atom molecular dynamics simulations.

**Availability and implementation:** The source code is freely available for download at http://www.cs.ox.ac.uk/mosaics/.

**Contact:** bernhard.knapp@stats.ox.ac.uk

**Supplementary information:** Supplementary data are available at *Bioinformatics* online.

## 1 Introduction

Presentation of protein fragments on the surface of antigen-presenting cells is a fundamental part of the human immune system. In virus-infected cells, proteosomes degrade viral proteins into peptides. These peptides are then transported via the transporter associated with antigen processing into the lumen of the endoplasmic reticulum where the peptides are loaded on major histocompatibility complex (MHC) class I molecules. These peptide/MHC (pMHC) complexes are then presented on the surface of antigen-presenting cells to the T-cell receptors (TCR) of T cells (Rudolph *et al.*, 2006). The linkage between MHC, peptide and TCR determines if an immune reaction is triggered against this peptide (van der Merwe and Dushek, 2011). Only if the binding affinity between peptide and MHC is strong enough, a peptide can be presented to TCR and a productive immune response against this peptide can take place. A wide range of pMHC binding affinity prediction methods have been developed (reviewed in Knapp *et al.*, 2009; Zhang *et al.*, 2012b). Sequence-based methods usually achieve high accuracy if sufficient training data are available. Structure-based methods are often worse at predicting binding affinity but give insight into the binding mode of the peptide (compare Knapp *et al.*, 2009; Zhang *et al.*, 2012b). Prediction methods based on molecular dynamics (MD) simulation attempt to not only predict the binding affinity and binding mode but also the dynamics of a peptide bound inside the MHC binding groove (Knapp *et al.*, 2015). However, no all-atom MD simulation has so far been able to give insight into the structural detachment process of a peptide from an MHC. This is likely to be due to the immense resource consumption that standard MD would take to map out the conformational space of pMHC detachment.

There are a large number of methods that have the potential to enhance sampling of structural simulations over standard MD. These include coarse graining at different levels [e.g. bond length constraints (Mazur, 1998), increased masses (Feenstra *et al.*, 1999), virtual sites (Hess *et al.*, 2008), *n*-bead models (Minary and Levitt, 2008) or the movement of rigid protein segments (Sim *et al.*, 2012)], biased force methods [e.g. metadynamics (Leone *et al.*, 2010), steered MD (Bayas *et al.*, 2003; Cuendet *et al.*, 2011; Kosztin *et al.*, 1999) or umbrella sampling (Torrie and Valleau, 1977)], replica exchange MD (Bernardi *et al.*, 2015), Monte Carlo simulations or alchemistic methods (Spiwok *et al.*, 2014). While these methods have been employed successfully for several ligand/receptor interactions, they have so far not been used to study pMHC detachment. Therefore, to date, neither experiments nor simulations have provided structural information on potential peptide detachment pathways from MHC.

In this study, we gain structural insight into the process of peptide detachment from the MHC HLA-A*02:01 by identifying a large number of low energy conformational states along the detachment pathway. Instead of all-atom MD, we use a simplified protein representation combined with generalized collective degrees of freedom and repeated simulated annealing. These three steps use already established methods: First, we coarse grain our all-atom model by using a 3-point-based amino acid representation (Minary and Levitt, 2008) and a knowledge-based statistical potential (Minary and Levitt, 2008). Second, we use hierarchical natural move Monte Carlo (HNMMC) (Sim *et al.*, 2012) to control the degrees of freedom. Third, we use temperature modulation (Zhang *et al.*, 2012a) to efficiently sample the energy landscape. On the basis of this protocol, we are for the first time able to give a comprehensive structural insight into the detachment processes of peptides from MHCs.

## 2 Methods

All-atom MD simulations are often hampered by two obstacles: the large number of degrees of freedom and the complexity of the energy function. In this study, we address these challenges by the combination of coarse graining, HNMMC and temperature annealing accelerated conformational optimization.

### 2.1 Coarse-grained protein model and force field

The all-atom pMHC structures were converted into 3-point representations (Minary and Levitt, 2008) using gro2mat (Dien *et al.*, 2014). In this representation, an amino acid is modelled by the α-carbon and carbonyl oxygen backbone atoms as well as a point at the centre of the side chain.

The previously established (Minary and Levitt, 2008) 3-point knowledge-based potential was used for the simulations. A coarse-grained potential can in principal allow atoms to approach closer than the excluded volume. To ensure that our small peptides did not approach the protein surface too closely, we uniformly scaled all pair interaction energies by the continuous non-linear function:

$$s(r)=\left\{ \begin{array}{l} s_{0}+(1-s_{0}){\left(\frac{r}{r_{0}}\right)}^{6}\;if\;r<r_{0}\\ 1.0\;if\;r\geq r_{0} \end{array}\right.$$

Where $r_{0}$ = 0.7 nm represents approximately the size of a large amino acid and $S_{0}$ = 0.15 was chosen, so that all peptides can escape deep energy minima. All pair interactions over 0.7 nm are identical to the established knowledge-based potential (Minary and Levitt, 2008).

### 2.2 Hierarchical natural move Monte Carlo

**Fig. 1.**
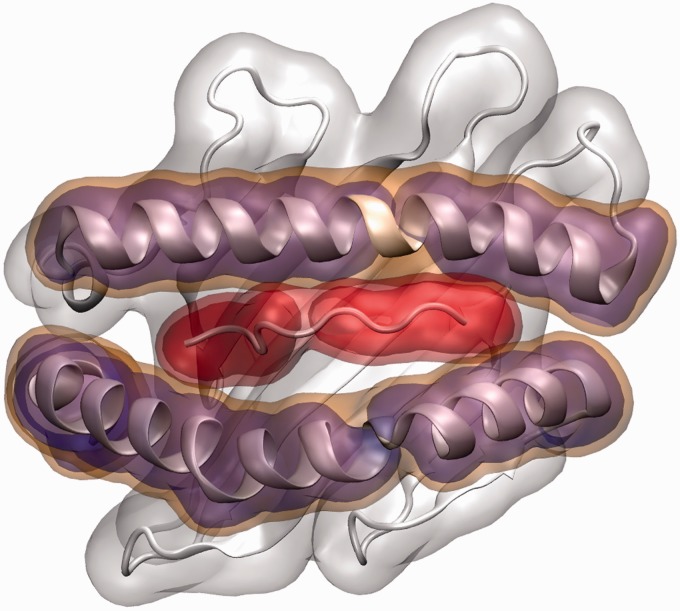
Structure of the pMHC complex HLA-A*02:01 based on PDB accession code 3PWN. The peptide is bound above the β-sheet floor and flanked by two kinked α-helices. The decomposition of the pMHC complex into regions as used in this study is illustrated with transparent surfaces. White: MHC β-floor; red: peptide; orange: whole MHC helices; magenta: MHC regions broken by kinks. For clarity, the α3 region and the β2-microglobulin are not shown

We follow the previously described HNMMC methodology (Sim *et al.*, 2012), which has been used in combination with the above coarse-grained model (Zhang *et al.*, 2012a) and is implemented in the software package MOSAICS. Natural moves are degrees of freedom that describe the collective motion of groups of residues (called regions). In proteins, this could be the movement of a stable secondary structure element such as an α-helix or β-sheet. Regions can additionally be grouped together and thereby form super-regions. In our study, we grouped the MHC into seven regions (Fig. 1; schematically in Supplementary Appendix Fig. S1). Both helices can move independently from the rest of the MHC and are flexible in themselves around the evolutionary conserved kinks (Wilman *et al.*, 2014) position in the middle of the helices. Also the peptide can be moved as a whole as well as in sub-regions. This decomposition enables all essential motions of the pMHC while keeping the number of degrees of freedom to a (necessary) minimum. In our Markov chain Monte Carlo (MCMC) simulation, each region as well as super-region (e.g. the two regions of one MHC helix) is propagated independently along three translational and three rotational degrees of freedom. The resulting chain breaks are resolved by an efficient stochastic chain-closure algorithm (Minary and Levitt, 2010).

### 2.3 Temperature modulation

In structural simulations, higher temperature allows for more flexibility, while a lower temperature hampers flexibility. In this study, we used repeated simulated annealing, which allows the rapid search for an ensemble of energetically favourable structural states along the peptide detachment process. It has been implemented by using a temperature modulation protocol (Zhang *et al.*, 2012a) as described by the function

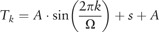



Where *A* is the amplitude of the temperature modulation, *k* the MCMC step counter, Ω is the number of steps per period and *s* is used to shift the minimum temperature. Similar to previous applications (Zhang *et al.*, 2012a), this conformational optimization protocol enabled the efficient exploration of important energy minima and corresponding pMHC conformations. In this study, we used *A* = 600 (Kelvin), *k* = 100 000, Ω = 5000 and *s* = 0 (Kelvin).

### 2.4 Preparation of the pMHC dataset

We used the MHC allele HLA-A*02:01 as it is among the most frequent MHC alleles in humans. To ensure that we did not use an outlier HLA-A*02:01 structure for our study, we extracted the 10 X-ray structures of HLA-A*02:01 with the highest resolutions from the protein data bank (PDB) (Berman *et al.*, 2000) and validated their amino acid sequence against the IMGT/HLA database (Robinson *et al.*, 2013). We then selected PDB-accession code 3PWN as it represents an average HLA-A*02:01 structure.

Thirty-two peptides with experimentally determined binding affinities were selected from Ishizuka *et al.* (2009) (Supplementary Appendix Table SI). We chose all peptides from the same study as this makes it likely that the measurements are comparable in rank order. These peptides were chosen to cover the whole range of observed experimental binding affinities. We chose a dataset with experimental IC50 values as those are available in abundance [e.g. from the Immune Epitope Data Base (IEDB) (Vita *et al.*, 2010)] and therefore used for benchmarking most pMHC binding affinity predictors.

SCWRL (Krivov *et al.*, 2009) and the peptX (Knapp *et al.*, 2011b) framework were used to model the 32 peptides of Ishizuka *et al*. (2009) into the MHC binding groove of PDB accession code 3PWN. This has been shown to be the most appropriate approach for altered pMHC modelling (Knapp *et al.*, 2008).

### 2.5 Performed simulations

**Fig. 2.**
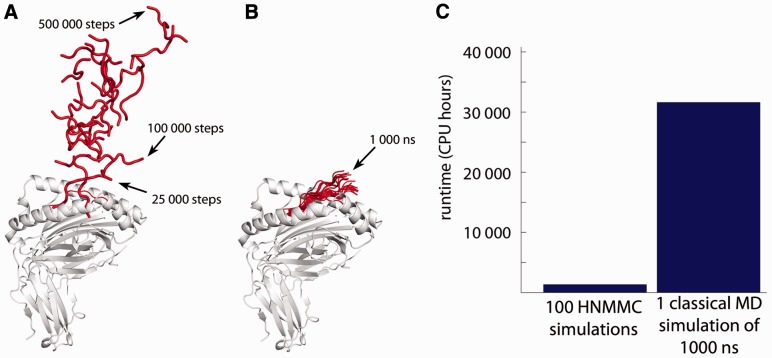
Simulated detachment process of AAAKTPVIV from HLA-A*02:01. (**A**) Equally distributed snapshots of the 500 000 HNMMC simulation steps. This simulation took about 13 h on a single core of an Intel i7-3770 3.40 GHz CPU. (**B**) Equally distributed snapshots of a 1000 ns MD simulation. The simulation took about 247 h using 128 Xeon cores at 2.0 GHz of the Oxford Advanced Research Computing facility. (**C**) Runtime comparison between our HNMMC simulations and a single MD simulation. The 1000 ns MD simulation has about the same runtime demand as all the HNMMC simulations (*n* = 3200) of our study combined

Initial test HNMMC simulations were run for 500 000 steps. These simulations showed that detachment usually takes place within 100 000 MCMC steps (Fig. 2A). Therefore, we ran the simulations of all 32 modelled pMHC complexes for 100 000 steps using the above-described HNMMC protocol. We repeated each simulation 100 times using different random seeds.

For comparison, we performed a 1000-ns standard MD simulation [GROMACS 4.5 (Pronk *et al.*, 2013) with the GROMOS96 53a6 force field (Oostenbrink *et al.*, 2004) and explicit simple point charge (SPC) water] of the same pMHC.

## 3 Results

### 3.1 HNMMC gives insights into the peptide detachment processes

In Figure 2A, we show a representative detachment process of the experimentally known non-binding peptide AAAKTPVIV. Using our HNMMC protocol, important conformational states for the partial peptide detachment process of this peptide can be located within 25 000 steps. These states imply that this peptide starts to detach C-terminally. All relevant conformational states for the full detachment are detected after 100 000 steps (arrow in Fig. 2A). This corresponds to approximately 2.5 h of simulation time on a standard desktop machine (all 500 000 steps took about 13 h).

For comparison, we performed a 1000-ns MD simulation of the same pMHC (Fig. 2B). To date, this is the longest reported MD simulation of pMHC and took about 247 h using 128 cores. The runtime of this single MD simulation corresponds roughly to the overall runtime of all (*n* = 3200) HNMMC simulations (100 independent simulations for 32 different peptide sequences) of this project (Fig. 2C). There is a high degree of similarity between the first 25 000 frames of the HNMMC detachment process and the MD simulation (compare Fig. 2A and B). Both simulations start their detachment process by an up and down flapping of peptide’s C-terminal end. However, during 1000 ns of MD, only this partial detachment can be observed (Fig. 2B). Thus, our HNMMC-based protocol is capable of accelerating calculations for pMHC detachment processes by several orders of magnitude.

### 3.2 Putative peptide detachment pathways

Having demonstrated that the methodology is able to simulate peptide detachment and that the results show agreement with classical methods, we then simulated 32 different peptides and repeated each simulation 100 times with different random initial seeds to initiate stochastically different trajectories.

**Fig. 3.**
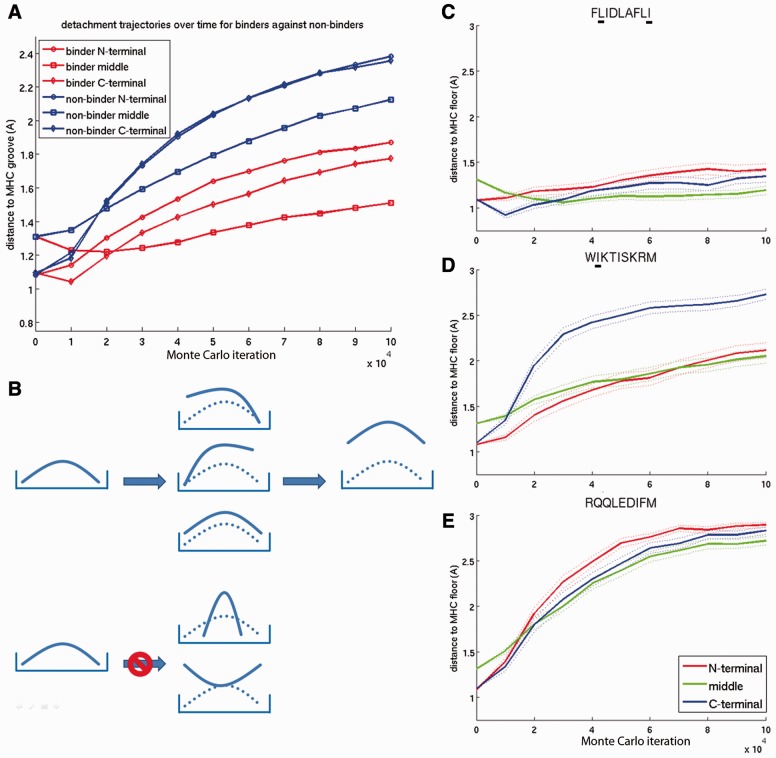
(**A**) Average peptide detachment trajectories split by experimentally known binders and non-binders. The same figure with error bars is shown in Supplementary Appendix Figure S3. (**B**) Schematic illustration of suggested peptide detachment pathways. Rectangular frame: MHC; bent solid line: peptide; bent dotted line: initial peptide configuration. Left column: initially, the peptide is bound in a slightly bent conformation within the MHC binding groove. Middle column: peptides most frequently start their detachment N- or C-terminally and in rare cases simultaneously from both sides. A detachment process starting from the middle or by bending the ends inversely was never observed. Right column: full detachment of the peptide is reached. (**C**) Stable binding between FLIDLAFLI and MHC due to matching anchor residues at peptide positions two and nine (**D**) Detachment pathway of the peptide WIKTISKRM from MHC. Peptide position two is a matching anchor residue. (**E**) Detachment pathway of the peptide RQQLEDIFM from MHC. This peptide contains no matching anchor residues. For (C–E), the average distance over 100 replicas is shown. The dotted lines indicate the standard error of the mean over the 100 replicas. Values above 3 nm were considered as full detachment and therefore set to 3 nm. The detachment trajectories of all peptides are shown in Supplementary Appendix Figure S2

The average peptide detachment pathway, grouped by experimentally known binder and non-binder, is illustrated in Figure 3A. X-ray structures show that peptides bind in the MHC groove in a slightly bent configuration. This allows for a closer proximity between the peptide ends and the MHC binding groove than between the peptide middle and the MHC binding groove. All 32 peptides have an initial distance between peptide and MHC floor of 1.31 nm for the peptide middle (Cα5peptide to Cα28MHC) and 1.08 nm and 1.09 nm for the peptide N- and C-terminal ends (Cα1peptide to Cα99MHC and Cα9peptide to Cα117MHC, respectively) (Fig. 3A). The peptides do not start their detachment process from the middle but from the N- or C-terminal end or from all peptide positions at the same time (Fig. 3B). After only 20 000 steps, the middle distance tends to have become the shortest distance. The peptides generally do not show a preference for an N- or C-terminal start of the detachment process from HLA-A*02:01 (Fig. 3A), but individual peptides do have preferences for N- or C-terminal detachment (Fig. 3C–E and Supplementary Appendix Fig. S2).

**Fig. 4.**
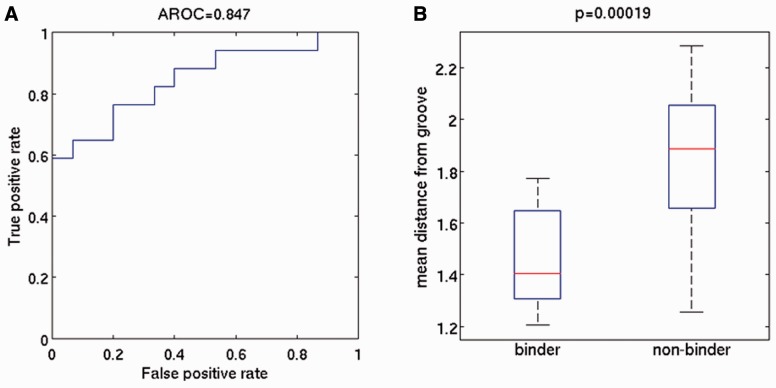
Reliability of HNMMC in comparison to experimental binding data. (**A**) ROC curve. (**B**) Boxplots of the average distance between MHC binding floor and the peptide for all simulations split by experimentally known binders and non-binders

Two mechanisms, which we never observed, are detachments starting from the peptide middle by either bending the ends inwards or bending the ends outwards (Fig. 3B, lowest panel).

### 3.3 The relationship between MHC anchor residues and peptide detachment pathways

The idea that certain residues are more important than others for MHC binding is called the anchor residue concept (Rammensee *et al.*, 1999). We investigated the relation between known anchor residues and the detachment pathways of our peptides. We extracted all experimentally tested HLA-A*02:01 peptides from the IEDB. In Supplementary Appendix Figure 4, we show the frequency of residues for binders and non-binders. This shows that binders have a preference for the hydrophobic residues L, M or I at peptide position 2 and V, L or I at peptide position nine. These residues are in agreement with the preferred anchors listed in the SYFPEITHI database (Rammensee *et al.*, 1999).

The presence or absence of these residues influences the detachment trajectories of the peptides. For example, the experimentally known binder FLIDLAFLI has anchor residue L at position 2 and anchor residue I at position 9 which keeps the peptide stable in the MHC binding groove for almost all replicas (Fig. 3C). The experimentally known non-binder WIKTISKRM has an anchor at peptide position 2 (I) but does not have an anchor residue in position nine. Therefore, its detachment process frequently starts C-terminally with the N-terminal end following later and more slowly (Fig. 3D). The experimentally known non-binder RQQLEDIFM does not have any matching anchor residues. It starts it detachment process simultaneously from both ends and the middle of the peptide (Fig. 3E).

This shows that anchor amino acids have a strong influence on the detachment trajectories of peptides.

### 3.4 Experimental binding affinity and peptide detachment

The accuracy of our HNMMC approach in discriminating between experimentally known MHC binders and non-binders gives an indication of the reliability of our proposed detachment trajectories. Non-binders should have larger distances to the MHC binding groove than binders i.e. they are likely to detach more quickly. We tested this by comparing the average distance over all replicas of a peptide against its experimentally known binding affinity. This test yields an area under the receiver operating characteristic curve (AROC) of 0.85 (Fig. 4A) and Pearson correlation coefficient of 0.67 (Supplementary Appendix Fig. S4). Furthermore, the difference between the pMHC-distances of all binders and all non-binders is significant (Fig. 4B).

**Fig. 5.**
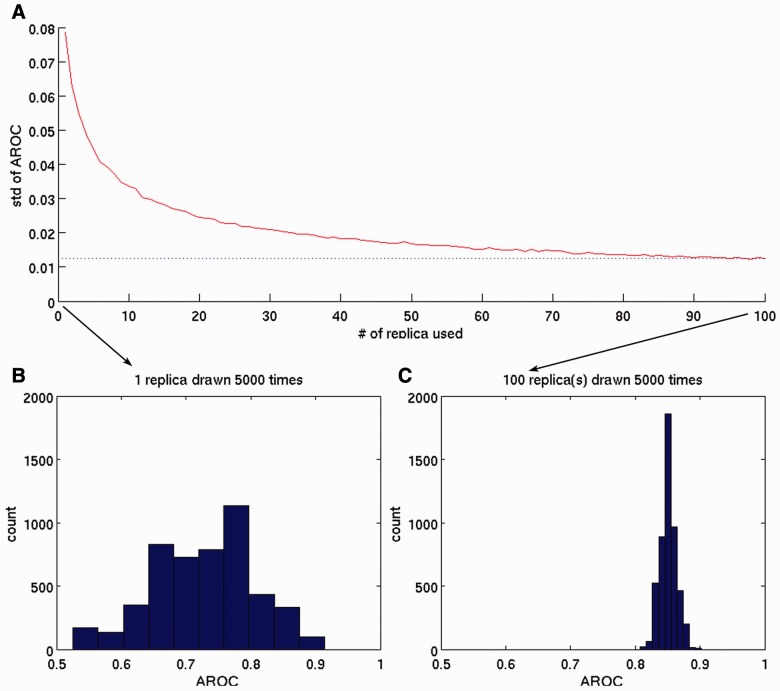
Bootstrapping analysis of replica numbers per peptide. (**A**) The standard deviation in the AROC between 5000 random selection procedures is shown against the number of replica used. (**B**) The distribution of the AROC of 1 replica per peptide chosen randomly 5000 times with repetition from our 100 replicas. The distribution ranges from 0.53 to 0.91. This shows that the results of a single simulation per peptide can lead to almost random results. The standard deviation of this distribution is shown as the first red dot of (A). (**C**) The same as (B) but for 100 replicas chosen randomly 5000 times with repetition from our 100 replicas. The ROC values of the 5000 boot strap runs are much more similar and therefore reliable. The standard deviation of this distribution is shown as the last red dot of (A)

Single simulations might be misleading because the conformational exploration could be trapped in one or few local minima. The use of multiple replica simulations is usually more reliable. To test whether this is the case for our pMHC detachment simulations, we performed a boot-strapping analysis using the 100 replicas per peptide. We investigated how the results would change if fewer replicas are taken into account. We randomly chose *n* (taking the values 1 to 100) replicas out of our 100 replicas with repetition. We calculated the AROC against experimental data. We repeated this 5000 times for each *n* and calculated the standard deviation between the 5000 AROC values. Each point in Figure 5A is the standard deviation over the 5000 AROC values. If only one replica is used, the standard deviation is 0.08 and the AROCs stretch between 0.53 (close to complete randomness) and 0.91 (close to perfect agreement). For 100 replicas, the standard deviation drops to 0.01 and the AROC values range only from 0.81 to 0.89 (Fig. 5B). Figure 5A shows a sharp descent of the AROC standard deviations until 25 replicas and a slower descent until 50 replicas.

This shows that our HNMMC approach can predict pMHC detachment processes with high accuracy and reliability if at least 25, if not 50, replicas are used.

## 4 Discussion

A large number of MD studies have investigated the structural interaction between peptide and MHC (reviewed in Knapp *et al.*, 2015). In none of these studies has full detachment of the peptide been observed. The longest reported pMHC MD simulation was 400 ns by (Narzi *et al.*, 2012). In this study, we ran a 1000-ns simulation of an experimentally known non-binding peptide in complex with MHC and observed only partial peptide detachment. This shows that current standard MD simulations are not giving insight into the pMHC detachment processes within a reasonable time frame. Consequently, most structural analysis has been carried out on bound pMHC (Hischenhuber *et al.*, 2012, 2013) and TCR/pMHC structures (Dunbar *et al.*, 2014; Knapp *et al.*, 2014) or empty MHC binding grooves (Rupp *et al.*, 2011; Yaneva *et al.*, 2009).

To obtain insight into the peptide detachment processes, we used the combination of three technologies. First, the coarse-grained 3-point model (Minary and Levitt, 2008) which allowed for a reasonable runtime while keeping specific features of the amino acid side chains. Second, hierarchical segmentation (Sim *et al.*, 2012) of the protein which further restricted the degrees of freedom and prevented global denaturation or local spoiling at high amplitudes. Third, repeated simulated annealing (Zhang *et al.*, 2012a) which allowed for an efficient sampling of low energy states along the energy surface. In this way, we were able to bypass the bottleneck of computational power and show how a detachment process of a peptide from MHC may occur.

There are alternative techniques to our ‘coarse grained hierarchical Monte Carlo simulated annealing approach’ that could also enhance the sampling (see introduction). However, to our knowledge, none of these methods have been employed for pMHC detachment yet.

Structural sampling methods will always produce a diverse collection of conformations of the structure under investigation. These movements are based on several parameter choices in the simulation setup. How meaningful these conformations are can be determined by comparison to experiments. Therefore, we compared our detachment data to two different types of experimental data.

First, we compared the detachment process with preferred anchor amino acids deduced from the IEDB (Zhang *et al.*, 2008). While anchor residues are not exclusively responsible for pMHC binding, we found frequent agreement between an N- or C-terminal detachment and the presence/absence of preferred anchor residues (Fig. 3C–E).

Second, we investigated if our detachment trajectories can discriminate between experimentally known binders and non-binders. We achieved high agreement (Fig. 4) between predicted detachment speed and experimental binding affinity data of (Ishizuka *et al.*, 2009). An AROC of 0.85 of our training-free approach is roughly in the range of sequence-trained pMHC binding prediction methods (Zhang *et al.*, 2012b) and superior to structural ligand/protein docking methods applied to pMHC (Knapp *et al.*, 2009).

These findings demonstrate that our coarse-grained HNMMC pMHC model is biophysically accurate and can capture the main factors contributing to the outcome of binding.

Even if methods such as temperature modulation (Zhang *et al.*, 2012a) or HNMMC (Sim *et al.*, 2012) are used, there is a finite probability that simulations will get trapped in local minima or run outlier trajectories leading to a questionable convergence (Knapp *et al.*, 2011a). Therefore, we decided to run a total of 100 replica simulations with different initial seeds per pMHC. On the basis of a boot strapping analysis, we found that about 25–50 replicas are needed for reliable conclusions. This is in agreement with recent studies that showed that the comparison between few MD simulations can yield to misleading results (Knapp *et al.*, 2014) and that 50 replicas are necessary for reliable binding free energy prediction of HIV drugs to HIV-1 Protease (Wright *et al.*, 2014) and peptides to MHC (Wan *et al.*, 2015).

## 5 Conclusion

In this study, we showed that HNMMC is able to give insight into the peptide detachment process from MHC. For the first time, we were able to analyse peptide detachment trajectories and thereby provide new views of the MHC structural landscape.

## Supplementary Material

Supplementary Data
