# Supplementary material for: Exploring peptide/MHC detachment processes using hierarchical natural move Monte Carlo
Source: Bioinformatics. 2015 Sep 22;32(2):181–6. doi: 10.1093/bioinformatics/btv502 (PMC4708099; doi:10.1093/bioinformatics/btv502)
Supplement: Supplementary Data [file supp_32_2_181__index.html]

Exploring peptide/MHC detachment processes using hierarchical natural move Monte Carlo — Exploring peptide/MHC detachment processes using hierarchical natural move Monte Carlo — Supplementary Data 

# Exploring peptide/MHC detachment processes using hierarchical natural move Monte Carlo

## Supplementary Data

files

- Supplementary Data - docx file
