# Supplementary material for: Exploring peptide/MHC detachment processes using hierarchical natural move Monte Carlo
Source: Bioinformatics. 2015 Sep 22;32(2):181–6. doi: 10.1093/bioinformatics/btv502 (PMC4708099; doi:10.1093/bioinformatics/btv502)
Supplement: Supplementary Data [file supp_btv502_supplementaryMaterial.docx]

**Supplementary Material:**

**
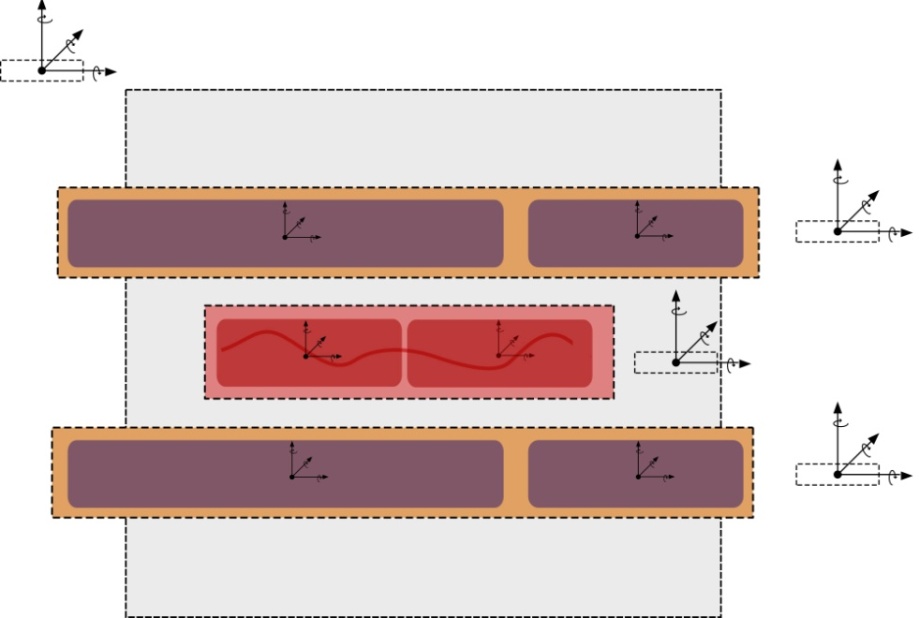
**

**Appendix Figure 1**: Schematic representation of the peptide/MHC regions used in this study. The axes indicate the six degrees of freedom per region.


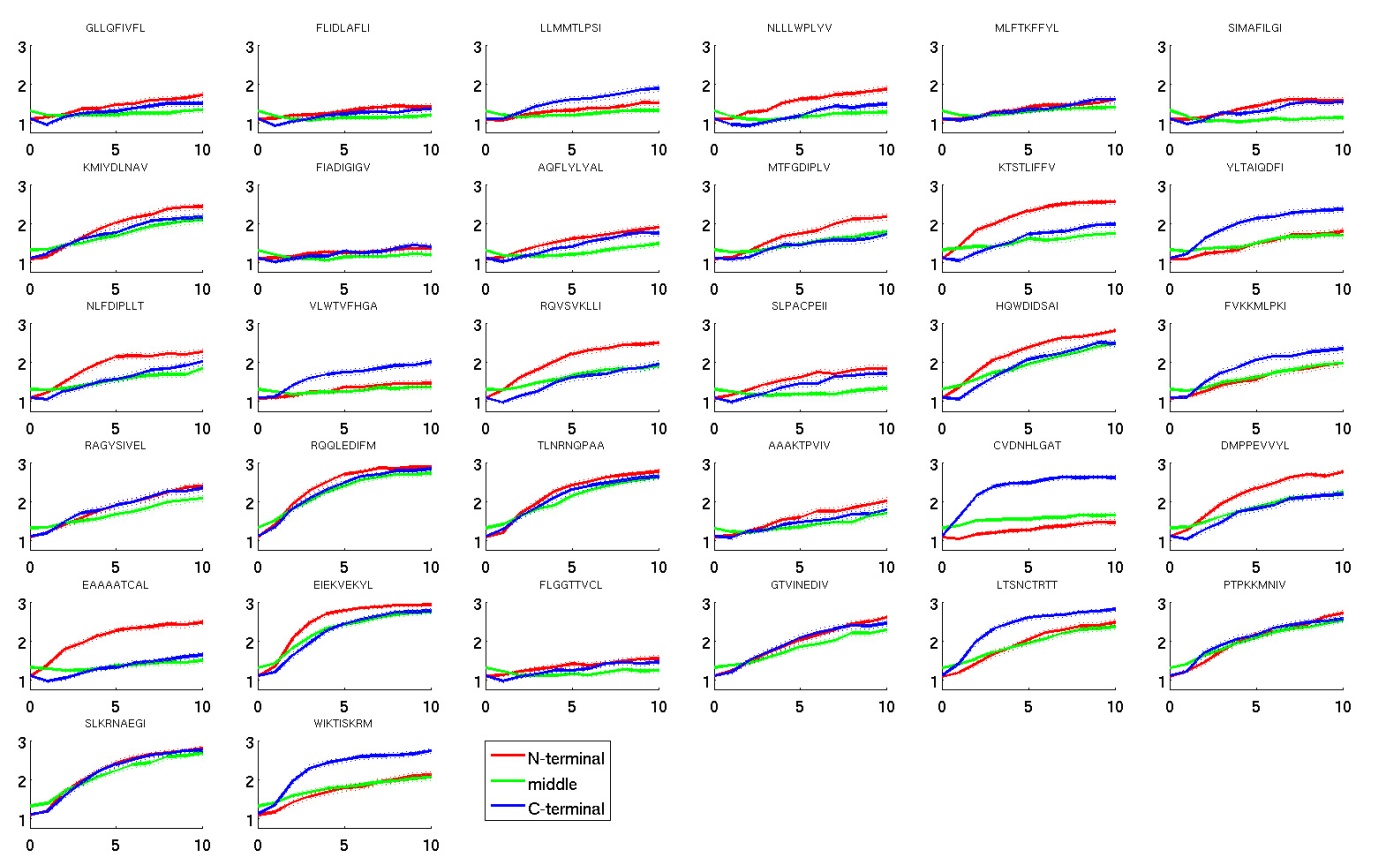


**Appendix Figure 2:** Detachment pathways for all 32 peptides. The pathways are averaged over the 100 replicas per peptide and the error bar indicates the standard error of mean.


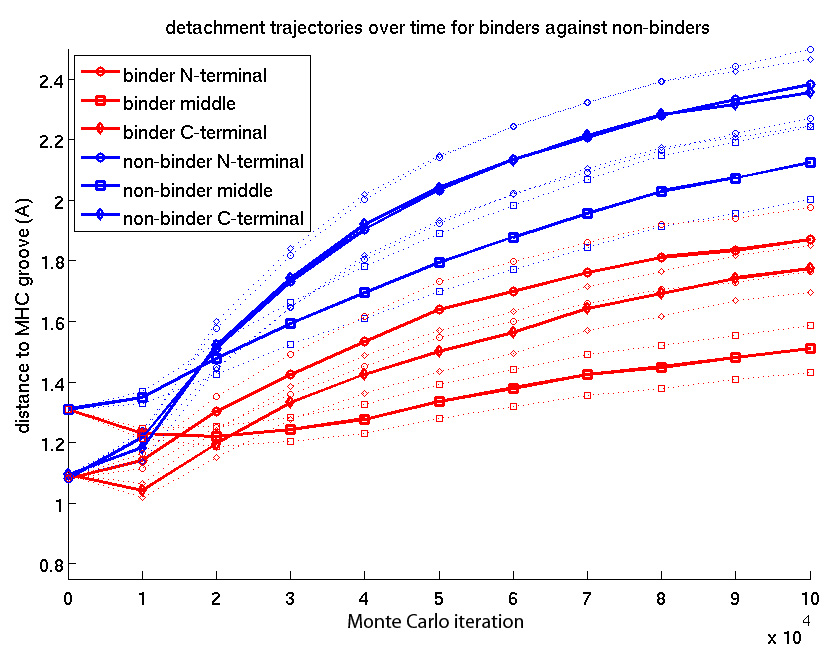


**Appendix Figure 3:** Same as Figure 3A but including the standard error of mean over the replicas.


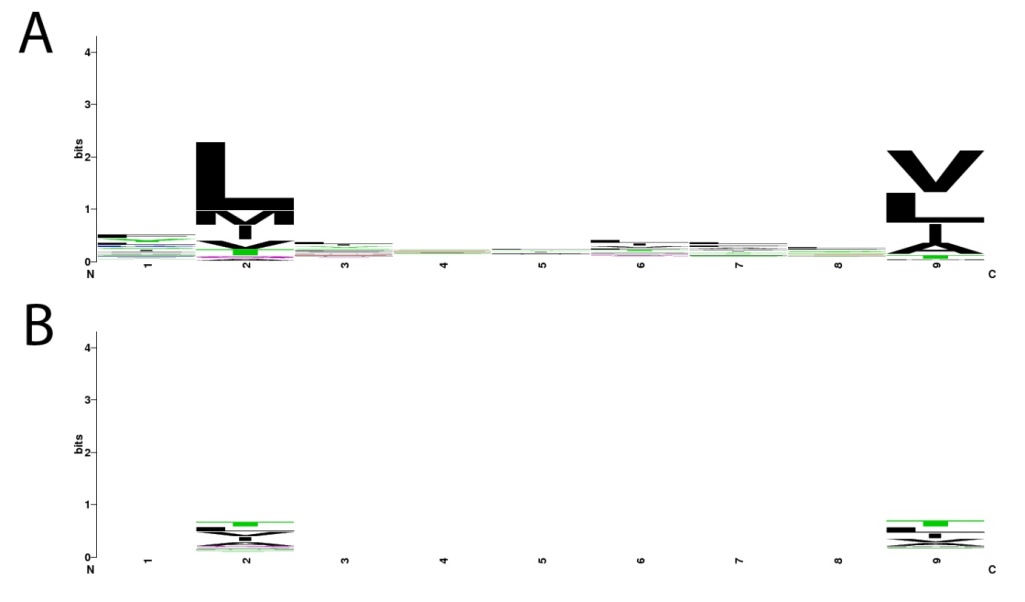


**Appendix Figure 4:** HLA-A*02:01 anchor residues. (A) Sequence logo (Crooks et al., 2004) based on 2266 experimentally known binder (IC50 ≤ 100 nM) from the IEDB. (B) Sequence logo based on 1598 experimentally known non-binder (IC50 ≥ 20000) from the IEDB.


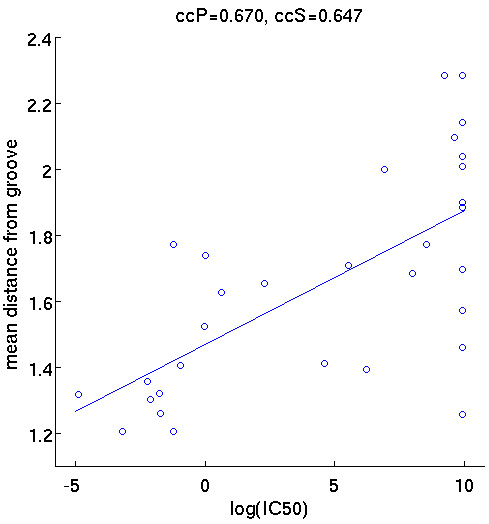


**Appendix Figure 5:** Correlation between detachment and experimentally measured binding affinity

| **Peptide** | **IC50 value** | |  | **Peptide** | **IC50 value** |
| --- | --- | --- | --- | --- | --- |
| AAAKTPVIV | 20000 |  | | LTSNCTRTT | 20000 |
| AQFLYLYAL | 0.4 |  | | MLFTKFFYL | 0.2 |
| CVDNHLGAT | 20000 |  | | MTFGDIPLV | 1 |
| DMPPEVVYL | 20000 |  | | NLFDIPLLT | 10 |
| EAAAATCAL | 20000 |  | | NLLLWPLYV | 0.1 |
| EIEKVEKYL | 20000 |  | | PTPKKMNIV | 20000 |
| FIADIGIGV | 0.3 |  | | RAGYSIVEL | 5000 |
| FLGGTTVCL | 20000 |  | | RQQLEDIFM | 10033 |
| FLIDLAFLI | 0 |  | | RQVSVKLLI | 250 |
| FVKKMLPKI | 3004 |  | | SIMAFILGI | 0.2 |
| GLLQFIVFL | 0 |  | | SLKRNAEGI | 20000 |
| GTVINEDIV | 20000 |  | | SLPACPEII | 504 |
| HQWDIDSAI | 1007 |  | | TLNRNQPAA | 15190 |
| KMIYDLNAV | 0.3 |  | | VLWTVFHGA | 100 |
| KTSTLIFFV | 1 |  | | WIKTISKRM | 20000 |
| LLMMTLPSI | 0.1 |  | | YLTAIQDFI | 1.9 |

**Appendix Table I:** Experimentally measured binding affinities of the 32 peptides of this study. The data is based on Ishizuka et al. (2009).
